# Ultrasound Imaging of Abdominal Wall Endometriosis: A Pictorial Review

**DOI:** 10.3390/diagnostics11040609

**Published:** 2021-03-29

**Authors:** Giulio Cocco, Andrea Delli Pizzi, Marco Scioscia, Vincenzo Ricci, Andrea Boccatonda, Matteo Candeloro, Marco Tana, Giuseppe Balconi, Marcello Romano, Cosima Schiavone

**Affiliations:** 1 Unit of Ultrasound in Internal Medicine, Department of Medicine and Science of Aging, “G. d’Annunzio” University, 66100 Chieti, Italy; andrea.boccatonda@gmail.com (A.B.); marco_tana@yahoo.it (M.T.); cosima.schiavone@gmail.com (C.S.); 2 Department of Neurosciences, Imaging and Clinical Sciences, “G. d’Annunzio” University, 66100 Chieti, Italy; andrea.dellipizzi@unich.it; 3 Unit of Gynecology Surgery, Mater Dei Hospital, 70125 Bari, Italy; marcoscioscia@gmail.com; 4 Physical and Rehabilitation Medicine Unit, “Luigi Sacco” University Hospital, A.S.S.T. Fatebenefratelli-Sacco, 20157 Milan, Italy; vincenzo.ricci58@gmail.com; 5 Department of Innovative Technologies in Medicine and Dentistry, University “G d’Annunzio”, 66100 Chieti, Italy; mat.candeloro@gmail.com; 6 Unità Operativa di Radiologia Diagnostica, Ospedale San Raffaele sede di Turro, 20127 Milan, Italy; gbalcon@tin.it; 7 Geriatrics Department, Garibaldi Hospital, 95123 Catania, Italy; marcelloromano@tin.it

**Keywords:** abdominal wall endometriosis, ultrasound, cesarean scar, gynecological surgical scar

## Abstract

Endometriosis is a debilitating disease characterized by endometrial glands and stroma outside the endometrial cavity. Abdominal wall endometriosis (AWE) indicates the presence of ectopic endometrium between the peritoneum and the skin, including subcutaneous adipose tissue and muscle layers, often following obstetric and gynecological surgical procedures. AWE is a not infrequent gynecological surgical complication, due to the increasing number of cesarean sections worldwide. In this pictorial review, we discuss the importance of medical history and physical examination, including the main ultrasound features in the diagnosis of AWE.

## 1. Introduction

Endometriosis is a debilitating disease characterized by the presence of endometrial glands and stroma outside the endometrial cavity. It is estimated to affect approximately 7–10% of women, with clinically relevant symptoms affecting approximately 3% of women of fertile age [1].

The ectopic tissue is responsive to ovarian hormonal stimulation and proliferates when stimulated by cyclic estrogens, thus appearing to “menstruate” [2]. This disease represents a public health problem, with a major effect on the quality of life of women, as well as having an economic burden [3].

In light of the considerable progress with diagnostic imaging (ultrasound and MRI), exploratory laparoscopy should no longer be used to diagnose endometriotic lesions [4]. Instead, the diagnosis of endometriosis should be based on a structured process involving a combination of patient interviews, clinical examination, and imaging [5,6]. This disorder affects women of reproductive age and generally occurs in pelvic sites, such as the ovaries, bowel, or pelvic peritoneum [7]. 

Extra-pelvic foci of ectopic endometrial tissue have been described in almost all organs and tissues of the body [8]. Medical history, together with a gynecological examination, has a combined sensitivity of around 80% for diagnosing endometriosis [9].

Abdominal wall endometriosis (AWE) indicates the presence of ectopic endometrium between the peritoneum and the skin, including subcutaneous adipose tissue and muscle layers [10]. Currently, the incidence of AWE following obstetric and gynecological procedures is higher due to the increasing number of caesarian sections worldwide [11]. In detail, the incidence of AWE is about 3.5% in patients who undergo gynecological surgery and is about 0.8% in all women with a previous cesarean section [12,13]. Imaging represents a mainstay of the diagnosis and follow-up of abdomino-pelvic diseases [14,15,16]. In this pictorial review, the authors provide a practical overview on abdominal ultrasound imaging of wall endometriosis, considering the cutaneous, umbilical, subcutaneous, intramuscular, and groin (inguinal canal) locations. 

## 2. Cutaneous Endometriosis 

Cutaneous endometriosis (CE) is known as the presence of endometrial tissues involving the skin. This condition is estimated to occur at approximately less than 1% of ectopic sites [17]. Generally, CE occurs secondarily to abdominal surgery, but can be primary, without any previous operations. Physical examination can reveal a palpable, tender, cutaneous mass on abdominal incision, sometimes with multiple tiny red orifices protruding from the skin. In addition to cyclical pain, the patient can show a brownish discharge from the lesion during menstruation. CE is often confused with other dermatological conditions (e.g., hypertrophic scar or keloid or infection of the scar), thus making the differential diagnosis difficult. [18]. 

## 3. Umbilical Endometriosis

**Figure 1:**
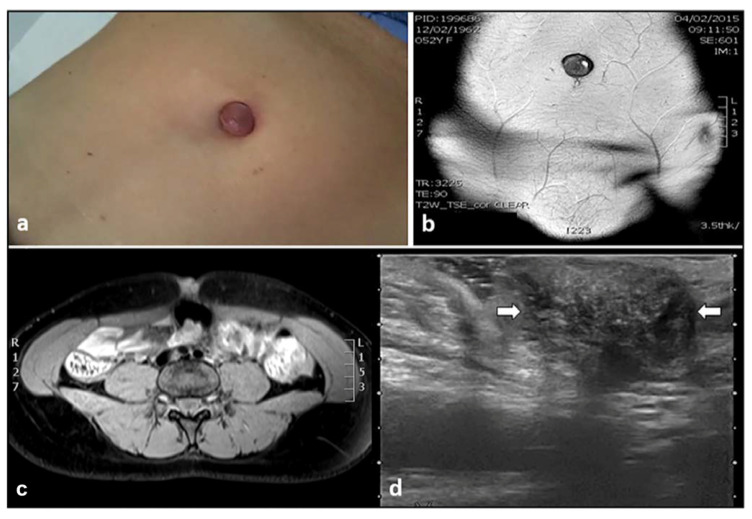
Umbilical mass, identified during physical examination (**a**), studied with MRI (**b**,**c**), and ultrasound imaging (**d**). White arrows in d revealed a hypoechoic hernial sac and intralesional hyperechoic spots, representing an umbilical hernia with endometriosis foci.

Umbilical endometriosis is a rare condition that accounts for only 0.5–1% of endometrial ectopy [19]. Umbilical endometriosis comprises 30–40% of all CE cases [20], and usually develops in a previous surgical scar (laparoscopic surgery or CS). In rare cases it can be primary [21]. The first case of umbilical endometriosis was credited in 1886 to Villar, hence the term “Villar node” [22]. Umbilical endometriosis can sometimes be associated with an umbilical hernia [19]. That condition is particularly facilitated by previous pregnancies and surgery. When people have undergone abdominal surgery (laparotomy or laparoscopic surgery), the incisions into the abdominal cavity may not heal well and a hernia can form in this site [19,20,21]. Umbilical endometriosis usually occurs in patients of reproductive age, is typically solitary, and presents a palpable nodule at the umbilicus. Moreover, it may coexist with pelvic endometriosis. The nodule can be covered by a reddish brown discharge, with swelling, pain, discharge, or cyclical bleeding from the umbilicus. These lesions are variable in size, are usually bluish-black in color and become painful, larger, and bleed around the time of menses [23,24]. Ultrasound shows a well-defined hypoechoic mass on the skin of the abdominal scar or at the umbilicus. Intralesional vascular spots can be detected on color Doppler examinations. Moreover, an umbilical endometriosis hernia, through a defect in the linea alba, can be observed (Figure 1a–d).

## 4. Subcutaneous Endometriosis

**Figure 2:**
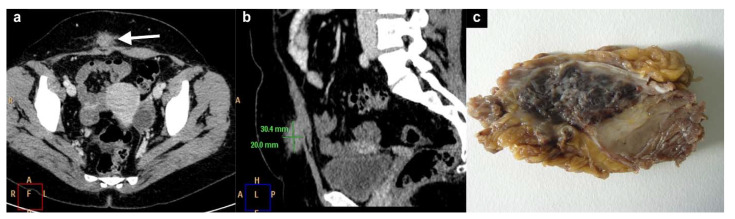
Axial (**a**) and Sagittal (**b**) CT images of a histologically proved (**c**) of subcutaneous endometriosis. A 30 × 20 mm^2^ endometriotic nodule (white arrow in a) was detected superficially and in contiguity with the right rectal muscle.

**Figure 3:**
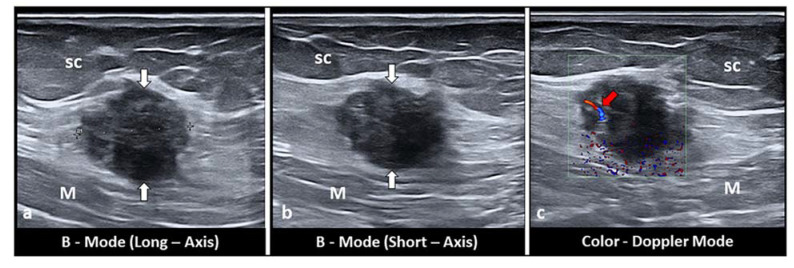
Long-axis (**a**) and short-axis (**b**) sonograms show a hypoechoic, nodular mass (white arrows), located between the subcutaneous tissue (sc) and the muscular plane (M) of the abdominal wall. Of note, fine intralesional, vascular spots (red arrow) are depicted on the color Doppler (**c**).

Subcutaneous endometriosis is the most frequent form occurring in the abdominal wall. Patients usually complain of focal tenderness in the right or left inguinal area, dyspareunia, with local swelling and a palpable mass [10,25,26,27]. If the woman is overweight, the mass may not be appreciated on palpation [27]. Generally, the past medical history is suggestive of cesarean or gynecological section. The abdominal pain can occur some years after the last cesarean section [10,13,25,27]. The nodule is located beneath the cesarean scar and superficially to the rectus abdominis muscle fascia (Figure 2a–c). Sonographic examination of the abdominal wall can reveal a heterogeneous hypoechoic mass with indistinct edges in the right or left inguinal region and at the level of the subcutaneous tissue. Color Doppler examination can detect the presence of intralesional vascular spots, and elastosonography can reveal a harder pattern compared to the surrounding tissues (Figure 3a–c) [27].

## 5. Intramuscular Endometriosis 

**Figure 4:**
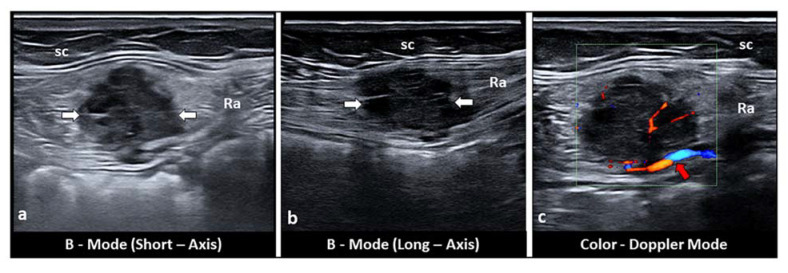
Short-axis (**a**) and long-axis (**b**) sonograms show a hypoechoic, nodular mass (white arrows), located inside the rectus abdominis (Ra) muscle. Of note, fine intralesional, vascular spots (red arrow) are depicted on the color Doppler mode (**c**). sc: subcutaneous tissue.

Intramuscular endometriosis lesions are often not palpable because the muscle tissue is deeper than the subcutaneous tissue [27]. Generally, the patient’s past medical history includes cesarean section or gynecological surgery. The abdominal pain, generally on the right or left side of the Pfannenstiel scar, can occur some years after the last cesarean section, with the pain being intermittent and strongly correlated with menstrual cycle [10,13,25,26,27]. Ultrasound examination can show a hypoechoic mass located inside the rectus abdominis muscle with indistinct edges, sometime with intralesional fluid collections. Color Doppler imaging may reveal the presence of intralesional vascular spots. Elastosonography usually shows a harder pattern compared to the surrounding tissues (Figure 4a–c).

## 6. Inguinal Canal Endometriosis

**Figure 5:**
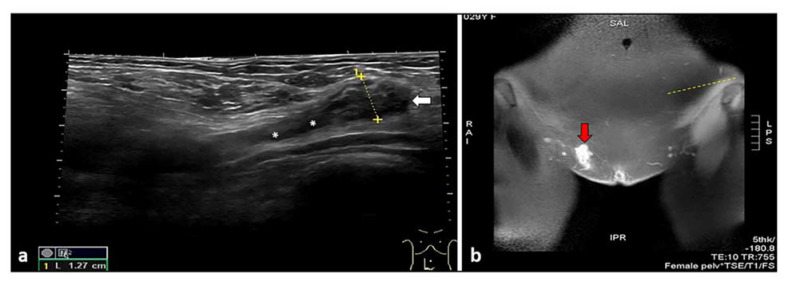
Extended field-of-view sonogram shows a hypoechoic and nodular mass (white arrow) located at the level of the Nuck canal (white asterisks) (**a**). Coronal T1-weighted MR image showing a hyperintense, pseudo-nodular mass (red arrow), confirming the presence of a hemorrhagic focus inside the inguinal canal representing endometriosis foci (**b**).

Inguinal hernia endometriosis is a possible cause of inguinal masses in females [28]. A hydrocele of the canal of Nuck in females is analogous to the encysted hydrocele of the spermatic cord in men [29,30]. Endometriosis localized to the canal of Nuck is very rare and difficult to appreciate clinically. The incidence of endometriosis in the extraperitoneal portion of the round ligament is 0.42% [31]. The patient often has a history of gynecological surgery or cesarean section, but this condition can also be primary. Generally, the pain pattern can be intermittent and strongly correlated with menstrual cycle. Ultrasound examination may show a hypoechoic solid nodule in the inguinal canal (Figure 5), but may contain a mixed solid and cystic component. In a recent review, Prodromidou et al. analyzed and evaluated the clinical presentation, diagnostic features, and management of Nuck’s endometriosis in 36 patients from 20 studies. The authors reported a median age of 36 years and a prevalence of right-side lesions [32].

## 7. Discussion

AWE is limited to the peritoneal surface and is the result of previous gynecological surgery [9,25,27,31,33]. Caesarean section represents the strongest risk factor for AWE considering the close contact that may commonly occur between endometrial cells and the subcutaneous tissue during this type of surgery [12,33]. Despite the already high incidence of AWE of about 0.8% in all women with a previous caesarean section, it is certainly underestimated [12,13]. Early diagnosis of AWE is difficult because it is not always clinically detectable [12,13]. Several studies have found that the time interval between surgery and clinical presentation is 3 months to 10 years [13,26,27]. The pathophysiological processes underlying AWE remain unclear [34,35,36,37,38]. AWE is believed to be the result of mechanical iatrogenic implantation, through the direct inoculation of the abdominal fascia and/or subcutaneous tissue with endometrial cells during the surgical intervention. In this way, it becomes active and expands under estrogen stimulation [22]. Some authors have examined factors that contribute to Caesarean section endometriosis and have defined some possible causes, including: the easy separation and transport of endometrial cells by the flow of amniotic fluid into the pelvic cavity after hysterotomy; the large amount of endometrial cells spreading into the pelvis before hysterotomy closure, which can become trapped in the wound; and the nurturing role of blood and hormones after inoculation of the cells, allowing them to grow and develop in subcutaneous tissue [31]. It is important to highlight that a high incidence is reported after early hysterotomy (end of the second or beginning of the third trimester), as the early decidua seems to have more pluripotent capabilities, potentially resulting in enhanced cellular replication producing endometriosis [26,27,28,29,30,31,33]. AWE is often misdiagnosed with several other pathological conditions, such as hernias, hematomas, vascular anomalies desmoid tumors, lymphomas, metastatic carcinomas, Sister Mary Joseph nodules, and sarcomas [10,25,38,39,40]. Although an abdominal solid mass detected at ultrasound cannot be immediately considered to be endometriosis, if the lesion is located close to the surgical section, AWE should be suspected [13]. However, endometriosis guidelines indicate that only histological examination can provide definitive confirmation of the diagnosis [9]. AWE can have a single focus or multiple foci and the location can be variable and widespread; the qualitative assessment of pain often shows a close relationship with the menstrual cycle, and this represents the main clue for the diagnosis of endometriosis [10,13,27]. Malignant transformation has been reported in 1% of endometriosis cases and mainly involves ovarian endometriosis (approximately 80%) [41,42]. There have been reports of malignant tumors occurring in AWE, although this transformation is quite rare and accounts for only 4.5% of all extra-genital neoplasms associated with endometriosis; in these cases, the most commonly reported histology is clear cell histology [41,42]. In a recent review, Ferrandina et al. described 23 cases of clear cell carcinoma resulting from Caesarean section endometriosis [43]. This cancer appears to be on the rise, probably due to greater knowledge of the disease and the increase in the rate of caesarean sections and uterine surgery in recent years [13,43]. The treatment of choice in all cases of abdominal wall endometriosis is a wide resection of the lesion, if necessary with partial resection of the underlying fascia. For most lesions, a margin of 1 cm is considered adequate [24].

**Figure 6:**
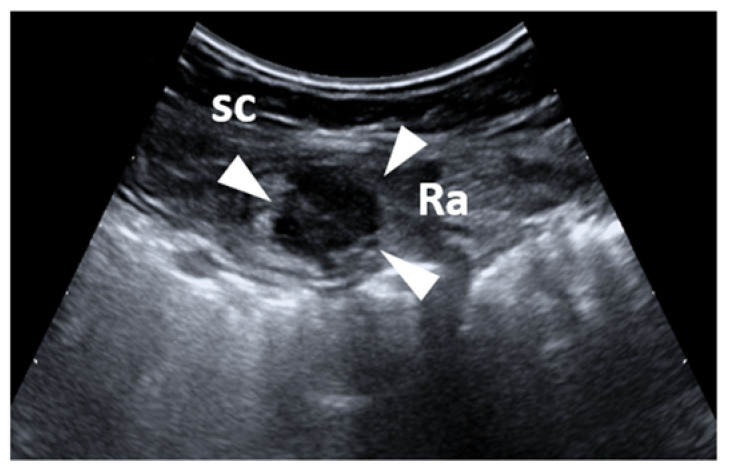
Short-axis sonogram with a convex transducer shows a hypoechoic, nodular mass (white arrow), located on the peritoneal superface, and inside the rectus abdominis (Ra) muscle. sc: subcutaneous tissue.

**Figure 7:**
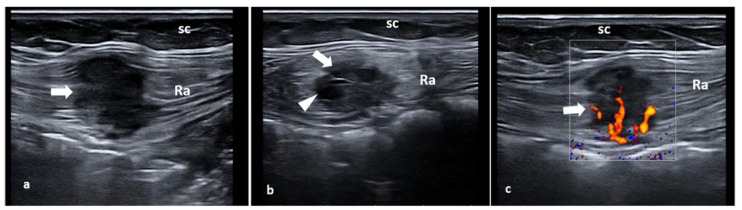
Long-axis sonograms show a hypoechoic, nodular mass inside the rectus abdominis with speculated borders (**a**). Short-axis sonograms show a partially cystic nodular (hypo-anechoic) mass with ill-defined margins (**b**). Fine intralesional vascular spots are depicted with color-power Doppler (**c**). sc: subcutaneous tissue ra: rectus abdominis muscle.

B-mode ultrasound images with a convex multifrequency transducer (2–8 MHz) can allow the identification of a non-specific nodule (often hypoechoic) with poor echo-structural details (Figure 6); moreover, small endometriotic lesions sometimes cannot be detected. The high-multifrequency linear transducer (3–13 MHz; 4–15 MHz; 8–24 MHz) can provide precise details for the surgeon, such as better definition of echostructural features and depths of the endometriotic lesion from the cutaneous plane. The endometriotic nodule may appear solid with variable echogenicity, usually hypoechoic or partially cystic (hypo- anechoic) with ill-defined margins or speculated borders; intralesional vascular spots are often depicted with color-power Doppler (Figure 7a–c) [13,43].

**Figure 8:**
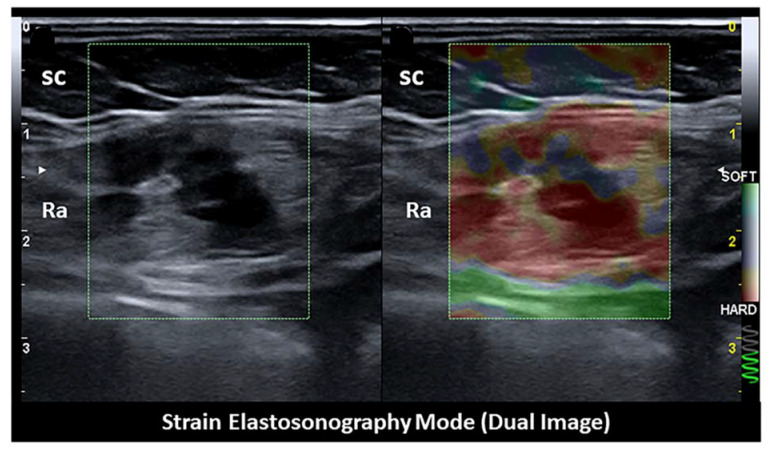
Strain elastosonography mode revealing the hard pattern of the endometriotic nodule. sc: subcutaneous tissue. ra: rectus abdominis muscle.

**Figure 9:**
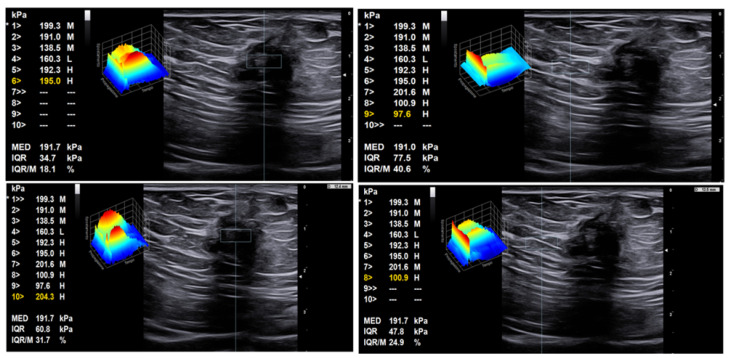
Share wave elastosonography mode revealing the hard pattern of the endometriotic nodule with respect to the surrounding tissue. Left: ROI within the tumor, with high shear wave pressures between 195 and 204 kPa; right: ROI within soft tissue surrounding the tumor, with distinctly lower shear wave pressures of 98–101 kPa.

Strain and share wave elastography may improve the characterization of lesions by highlighting the fibrotic component in endometriotic tissue. Indeed, several cell types, including activated platelets, macrophages, ectopic endometrial cells, and sensory nerve fibers, contribute to the development of fibrosis [44,45]. Elastography may be particularly useful if the lesion is isoechoic with respect to the surrounding tissue. In fact, strain and share wave elastography can detect a different stiffness pattern of endometrial lesion with respect to the surrounding tissue, thus confirming the ectopic nature of the masses (Figure 8 and Figure 9) [27]. Magnetic resonance is the method of choice to accurately define the involvement of different anatomical structures and diagnose the presence of deep endometriosis [46,47].

## 8. Therapeutic Options

Surgical excision is the treatment of choice in AWE. A wide incision for endometriotic nodules is recommended due to the risk of recurrence in 5–9% of cases [48,49]. For most lesions, a margin of 1 cm is considered adequate [24].

In select cases (e.g., in patients having high surgical risk or refusing surgical intervention) minimally invasive or conservative treatments can be proposed: sclerotherapy, high-intensity focused ultrasound ablation (HIFUA), or cryoablation and combined oral contraceptives [48]. Sclerotherapy with ultrasound guided ethanol injection into the scar endometriosis has been reported to be effective in some cases [50]. Recently, some authors have suggested HIFUA, but data are still scarce [51,52,53]. Crioablation has an emerging role in select patients with AWE [54]. Recently, case reports and small case series have described successful US or CT guided thermal ablation of AWE with durable symptomatic relief [55,56]. This technique is performed with argon-based cryoablation systems using cryoprobes [54]. Although from few studies, a clinical improvement with conservative treatment combining oral contraceptive progestogen and hormone suppression therapy with gonadotropin-releasing hormone (GnRH) analogues was also reported [48].

## 9. Conclusions

AWE following obstetric and gynecological surgery is becoming more frequently diagnosed due to the increasing number of cesarean sections worldwide. AWE should be suspected in any woman of childbearing age with a lump in the incisional scar area after pelvic surgery and pain that is strongly correlated to her menstrual cycle. AWE may be difficult to diagnose, especially if the mass is not palpable. Ultrasound imaging by a high multifrequency linear transducer is a precious tool to identify and characterize endometriotic lesions in the superficial tissues of the abdominal wall.
